# Modelling the impact of raising tobacco taxes on public health and finance

**DOI:** 10.2471/BLT.15.164707

**Published:** 2016-02-12

**Authors:** Mark Goodchild, Anne-Marie Perucic, Nigar Nargis

**Affiliations:** a Department for Prevention of Noncommunicable Diseases, World Health Organization, 20 avenue Appia, 1211 Geneva 27, Switzerland.; b American Cancer Society, Washington DC, United States of America.

## Abstract

**Objective:**

To investigate the potential for tobacco tax to contribute to the *2030 agenda for sustainable development *by reducing tobacco use, saving lives and generating tax revenues.

**Methods:**

A model of the global cigarette market in 2014 – developed using data for 181 countries – was used to quantify the impact of raising cigarette excise in each country by one international dollar (I$) per 20-cigarette pack. All currencies were converted into I$ using purchasing power parity exchange rates. The results were summarized by income group and region.

**Findings:**

According to our model, the tax increase would lead the mean retail price of cigarettes to increase by 42% – from 3.20 to 4.55 I$ per 20-cigarette pack. The prevalence of daily smoking would fall by 9% – from 14.1% to 12.9% of adults – resulting in 66 million fewer smokers and 15 million fewer smoking-attributable deaths among the adults who were alive in 2014. Cigarette excise revenue would increase by 47% – from 402 billion to 593 billion I$ – giving an extra 190 billion I$s in revenue. This, in turn, could help create the fiscal space required to finance development priorities. For example, if the extra revenue was allocated to health budgets, public expenditure on health could increase by 4% globally.

**Conclusion:**

Tobacco taxation can prevent millions of smoking-attributable deaths throughout the world and contribute to achieving the sustainable development goals. There is also potential for tobacco taxation to create the fiscal space needed to finance development, particularly in low- and middle-income countries.

## Résumé

**Objectif:**

Examiner la manière dont les taxes sur le tabac pourraient contribuer au Programme de développement durable pour 2030 en réduisant la consommation de tabac, en sauvant des vies et en générant des recettes fiscales.

**Méthodes:**

Un modèle du marché mondial des cigarettes en 2014 – élaboré à partir des données de 181 pays – a été utilisé pour quantifier l'impact de la hausse de l'accise sur les cigarettes d'un dollar international ($I) par paquet de 20 cigarettes dans chaque pays. Toutes les devises ont été converties en $I à l’aide de taux de change assurant la parité des pouvoirs d'achat. Les résultats ont été synthétisés par tranche de revenu et par région.

**Résultats:**

D'après notre modèle, cette hausse des taxes ferait augmenter le prix de détail moyen des cigarettes de 42% – de 3,20 à 4,55 $I par paquet de 20 cigarettes. La prévalence du tabagisme quotidien baisserait de 9% – passant de 14,1% à 12,9% des adultes – et l'on observerait 66 millions de fumeurs en moins et 15 millions de morts attribuables au tabac en moins chez les adultes qui étaient vivants en 2014. Les produits des droits d'accise sur les cigarettes augmenteraient de 47% – passant de 402 milliards à 593 milliards de $I – soit 190 milliards de $I de recettes supplémentaires. Cela pourrait offrir la marge de manœuvre budgétaire nécessaire au financement des priorités de développement. Si, par exemple, ces recettes supplémentaires étaient affectées aux budgets de la santé, les dépenses publiques dans ce domaine pourraient augmenter de 4% au niveau mondial.

**Conclusion:**

Les taxes sur le tabac permettent d'éviter des millions de morts attribuables au tabac dans le monde et contribuent à la réalisation des objectifs de développement durable. Elles pourraient également offrir la marge de manœuvre budgétaire nécessaire au financement du développement, en particulier dans les pays à revenu faible et intermédiaire.

## Resumen

**Objetivo:**

Investigar el potencial del impuesto sobre el tabaco de contribuir a la Agenda 2030 para el Desarrollo Sostenible mediante la reducción del consumo de tabaco, salvando vidas y generando ingresos fiscales.

**Métodos:**

Se utilizó un modelo del mercado global de cigarrillos en 2014 (desarrollado a partir de datos recopilados en 181 países) para cuantificar el impacto del aumento del consumo de cigarrillos en cada país por un dólar internacional (I$) por cada paquete de 20 cigarrillos. Se convirtieron todas las divisas en I$ mediante tipos de cambio de *paridad del poder adquisitivo*. Los resultados se resumieron por grupos de ingresos y por regiones.

**Resultados:**

Según nuestro modelo, el aumento de los impuestos daría lugar a una subida del precio minorista principal de los cigarrillos de un 42% (de 3,20 I$ a 4,55 I$ por cada paquete de 20 cigarrillos). La frecuencia del consumo de tabaco diario caería un 9% (de un 14,1% a un 12,9% de los adultos), lo que se traduce en 66 millones menos de fumadores y 15 millones menos de fallecimientos a causa del tabaco en adultos vivos en 2014. Los ingresos obtenidos del consumo de cigarrillos aumentarían un 47% (de 402 000 millones de I$ a 593 000 millones de I$), lo que ofrece unos ingresos adicionales de 190 000 millones de I$. Por su parte, esto ayudaría a crear el espacio fiscal necesario para financiar las prioridades de desarrollo. Por ejemplo, si los ingresos adicionales se asignaran a presupuestos sanitarios, el gasto público en salud aumentaría un 4% a nivel mundial.

**Conclusión:**

Los impuestos sobre el tabaco pueden evitar millones de fallecimientos atribuibles al tabaco en todo el mundo y pueden contribuir a lograr los objetivos de desarrollo sostenible. También existe la posibilidad de que los impuestos sobre el tabaco generen el espacio fiscal necesario para financiar el desarrollo, principalmente en países con ingresos bajos y medios.

## ملخص

**الغرض:**

استقصاء احتمال ****إسهام الضرائب المفروضة على التبغ في تنفيذ جدول الأعمال لعام 2030 الذي يهدف لتحقيق التنمية المستدامة عن طريق خفض معدل استهلاك التبغ، مما يحافظ على الأرواح ويدر إيرادات ضريبية.

**الطريقة:**

تم استخدام نموذج لسوق السجائر العالمية في عام 2014 – والذي تم وضعه بالاستعانة بالبيانات المتعلقة بعدد من الدول يبلغ 181 دولة – لقياس مدى أثر رفع الضرائب المفروضة على مبيعات السجائر بقيمة واحد دولار دولي (I$) لكل علبة سجائر تحتوي على 20 سيجارة في كل دولة على حدة. وتم تحويل جميع العملات إلى الدولار الدولي باستخدام أسعار صرف تعادل القوة الشرائية. كما تم إيجاز النتائج حسب فئة الدخل والمنطقة.

**النتائج:**

وفقًا للنموذج الذي استخدمناه، فإن الزيادة في الضرائب من المفترض أن تؤدي إلى الزيادة في متوسط سعر البيع بالتجزئة الخاص بالسجائر بنسبة ‏42‏% – من 3.20 إلى 4.55 دولار دولي لكل علبة سجائر تحتوي على 20 سيجارة. ويُفترض انخفاض نسبة انتشار التدخين اليومي بنسبة 9‏% – لتهبط بذلك من 14.1‏% إلى ‏12.9‏% بين الراشدين – مما يؤدي إلى الانخفاض بمعدل 66 مليونًا في أعداد المدخنين وبمعدل 15 مليونًا في حالات الوفاة التي تُعزى إلى التدخين بين الراشدين ممن كانوا على قيد الحياة في عام 2014، ومن المفترض زيادة الإيراد الضريبي للسجائر بنسبة ‏47‏% – من 402 مليار إلى 593 مليار دولار دولي – مما يتيح 190 مليار دولار دولي إضافي في الإيرادات. وقد يؤدي هذا الأمر بدوره إلى إيجاد الحيز المالي المطلوب لتمويل أولويات التنمية. ومثالاً لذلك، إذا تم تخصيص الإيرادات الإضافية للميزانيات المخصصة لقطاع الصحة، فمن الممكن أن يزيد الإنفاق العام على الصحة بنسبة 4‏% على الصعيد العالمي.

**الاستنتاج:**

يمكن أن تمنع الضرائب المفروضة على التبغ وقوع الملايين من حالات الوفاة التي تعزو إلى التدخين في أنحاء العالم، ومن الممكن أيضًا أن تسهم في تحقيق أهداف التنمية المستدامة. ويظهر أيضًا احتمالاً بأن تؤدي الضرائب المفروضة على التبغ إلى إيجاد الحيز المالي اللازم للتطوير التمويلي، خاصةً في الدول الأقل دخلاً والمتوسطة الدخل.

## 摘要

**目的:**

旨在调查通过减少烟草使用，拯救生命及征收税收，烟草税对 2030 年可持续发展议程的贡献可能性。

**方法:**

2014 年全球烟草市场模型 – 利用 181 个国家构建 – 被用于量化提升各国烟草消费税（即每 20 支包装增加 1 国际美元 (I$)）的影响。所有货币均按照购买力平价汇率转换成 I$。得出的结果根据工薪阶层和地区进行归纳。

**结果:**

根据我们的模型，税收增加将导致烟草的平均零售价格增加 42% – 从每 20 支包装 3.20 增加至 4.55 I$。成人每日吸烟的普遍性将降低 9% – 从 14.1% 降至 12.9% – 从而导致减少 6600 万烟民及减少 1500 万（2014 年存活的成人中）因吸烟导致的死亡。烟草消费税收入将增加 47% – 从 4020 亿增加至 5930 亿 I$ – 从而使得收入增加 1900 亿 I$。这反过来也有助于创造所需的财政空间，从而为发展重点筹资。例如，如果将额外收入分配至保健预算中，全球保健公共开支将增加 4%。

**结论:**

烟草税可预防全球近百万因吸烟导致的死亡，并有助于实现可持续发展目标。烟草税可能有助于创造财政空间，从而为发展筹资，尤其为中低收入国家。

## Резюме

**Цель:**

Изучить, какие возможности создает налогообложение табачной продукции в плане достижения целей, предусмотренных повесткой дня в области устойчивого развития на период до 2030 года, путем снижения потребления табака, уменьшения числа смертей и получения прибыли от налогов.

**Методы:**

С помощью данных по 181 стране была разработана модель мирового рынка сигарет в 2014 году, чтобы количественно измерить эффект от повышения акцизов на сигареты на один международный доллар (м. долл.) за пачку (20 сигарет) в каждой стране. Все валюты конвертировали в м. долл., используя обменные курсы, рассчитанные на основе паритета покупательной способности. Результаты были представлены по регионам и группам по уровню доходов.

**Результаты:**

Как показала выбранная модель, повышение налогов привело бы к 42-процентному повышению средней розничной цены на сигареты — с 3,20 до 4,55 м. долл. за пачку, содержащую 20 сигарет. Распространенность ежедневного курения снизилась бы на 9% — с 14,1 до 12,9% взрослых, в результате чего число курильщиков сократилось бы на 66 миллионов, а число смертей, обусловленных курением, — на 15 миллионов среди взрослого населения, жившего в 2014 году. Доход от акцизов на сигареты увеличился бы на 47% (с 402 до 593 млрд м. долл.), принеся 190 млрд м. долл. дополнительного дохода. Это, в свою очередь, могло бы создать возможности для бюджетного маневра, которые требуются для финансирования приоритетов развития. Например, если эта дополнительная прибыль была бы направлена в бюджет здравоохранения, государственные расходы на здравоохранение во всем мире увеличились бы на 4%.

**Вывод:**

Введение налога на табак может позволить избежать миллионы смертей, обусловленных курением, во всем мире и поспособствовать достижению целей устойчивого развития. Налогообложение табачных изделий также может создать возможности для финансирования развития, в частности в странах с низким и средним уровнями доходов.

## Introduction

Although the use of a tobacco tax to reduce smoking is still relatively new in many countries, there is a long history throughout the world of governments implementing such a tax to generate revenue. In many forums, tobacco taxation has also been highlighted as a means of mobilizing domestic resources to finance health and other development programmes.^1^^–^^3^ The recent setting of the Addis Ababa Action Agenda^4^ and the 2030 Agenda for Sustainable Development^5^ have further heightened interest in tobacco taxation.

In July 2015 the United Nations General Assembly endorsed the Addis Ababa Action Agenda. In this agenda, which was an outcome of the Third International Conference on Financing for Development, the United Nations recognized that “price and tax measures on tobacco can be an effective and important means to reduce tobacco consumption and health-care costs, and represent a revenue stream for financing for development in many countries.”^4^

Subsequently, in September 2015, the 2030 Agenda for Sustainable Development was also adopted in a United Nations General Assembly.^5^ This agenda includes 17 sustainable development goals (SDGs) that all Member States have agreed to achieve by 2030. SDG 3, which is to “ensure healthy lives and promote well-being for all ages”, includes target 3.4 – to reduce premature mortality from noncommunicable diseases by one third – and target 3.a – to strengthen country-level implementation of the World Health Organization’s (WHO’s) Framework Convention on Tobacco Control (FCTC).^5^^,^^6^

The FCTC is an international treaty with 180 Parties who have committed to protecting public health through the implementation of comprehensive measures of tobacco control. Article 6 of the FCTC recognizes price and tax measures as effective means to reduce the demand for tobacco, and the guidelines for Article 6’s implementation encourage the use of taxation in comprehensive strategies for tobacco control.^7^^,^^8^

It seems likely that tobacco taxation will be an important evidence-based intervention to help many countries achieve their development objectives. As tobacco tax rates in many low- and middle-income countries are currently low and demand for tobacco products is relatively inelastic, many countries could increase government revenues substantially through tobacco taxation.^9^ By creating the fiscal space to finance development programmes while, at the same time, reducing tobacco use, tobacco taxation could be a win-win policy for governments.

In the first year that the so-called sin-tax reforms were implemented in the Philippines, the tax on low-priced brands of cigarettes was increased by 341% and this led to a 114% increase in annual excise revenue.^10^ Under the reforms, 85% of the extra revenue is being used to subsidize universal health care for 14 million families and upgrade medical facilities. There are at least 30 other countries that dedicate a certain amount of their tobacco taxes to health.^10^ Although such dedicated allocations are not always feasible, the reforms in the Philippines have shown that substantial increases in tobacco taxation can lead to improvements in public health finance.

Retrospective studies have shown the importance of tobacco taxation in public health outcomes. For example, in the United States of America, it has been observed that a 10% increase in cigarette taxes could decrease the number of deaths from respiratory cancers by 1.5%.^11^ The French Government increased cigarette taxes substantially from the mid-1990s, with cigarette prices tripling in real terms by 2005. Among French males, rates of death from lung cancer fell by 50% during the same period.^12^^,^^13^

We wished to demonstrate the potential for tobacco taxation to reduce tobacco use, save lives and generate tax revenues globally. We therefore developed a model of the global cigarette market using data for 181 countries that together represented 98% of the world’s smokers. We used the model to quantify the impacts of the increase in excise on the retail price of cigarettes, cigarette excise revenue, cigarette consumption, the number of daily cigarette smokers, and the future number of smoking-attributable deaths averted among the world’s adult population in 2014.

## Methods

### Data sources

Data on taxes and prices per 20-cigarette pack of the most popular brand of cigarette in each study country in 2014 were sourced from WHO’s *Report on the global tobacco epidemic*.^10^ In this data set, the amount of excise and other taxes on cigarettes is calculated on the basis of each country’s actual tax system. Excise is a tax imposed on selected commodities such as cigarettes and is the main fiscal instrument that governments use to generate extra tax revenue from those commodities.

The quantity of cigarettes sold in each country was calculated using data from two market survey companies – Canadean^14^ and Euromonitor International^15^ – and from WHO’s work with Member States.^16^ The numbers of daily cigarette smokers were calculated using the United Nations Population Division’s country-specific estimates of the adult population in 2014 and WHO’s estimates of the prevalence of daily cigarette smoking among adults.^17^^,^^18^

Online databases of the International Monetary Fund, the World Bank and WHO were used to source macro-economic data on inflation, government health expenditure and purchasing power parity exchange rates.^19^^,^^20^ Our findings are reported in international dollars (I$) to provide an accurate comparison of cigarette prices between countries – after taking into account differences in the purchasing power of countries at different levels of income and development.

### Taxes and prices

Countries apply different kinds of excise systems to cigarettes. Excise may be a fixed amount per pack or a percentage of the pack’s value or a combination of the two. For each included country, the database of WHO’s *Report on the global tobacco epidemic* reports the amount of taxes on the most popular brand of cigarette. We took these amounts as the baseline levels of tobacco tax and then simulated the effects of increasing excise, by I$ 1.00 per 20-cigarette tax, over the next year. This level of intervention was chosen because it reduces the affordability of cigarettes in all Member States, particularly in low- and middle-income countries where cigarette taxes and prices are relatively low.^9^^,^^10^

We allowed general consumption taxes – e.g. value-added tax or sales tax – to rise as normal, on the basis of the retail or wholesale prices of the cigarettes. Although we fixed the per-pack values of other kinds of taxes – e.g. import duties or surcharges – this simplification would have had little impact on our main findings since excise and value-added tax are the most important taxes on cigarettes in most countries.

The retail price that consumers pay includes all applicable taxes plus the producer or industry price net of taxes. The retail price of a pack of cigarettes, *P_R_*, can be calculated as: *P_p_* + *T_E_* + *T_VAT_* +*T_O_*  (1) where *P_p_* is the producer price net of taxes, *T_E_* is the excise amount per pack, *T_VAT_* is the amount of value-added tax per pack and *T_O_* is the other taxes – e.g. import duties.

For the model, we used the standard assumption of full pass through of taxes onto the retail price of cigarettes.^21^ In addition, the producer price net of tax was assumed to increase in line with the global inflation rate – reflecting, for example, the maintenance of industry cost and profit margins in real terms. The new retail price that we modelled, P_R_*, was calculated as: P_p_* + T_E_* + T_VAT_* +*T_O_*  (2) where P_p_* is the new industry price per pack after adjusting for inflation, T_E_* is the new excise amount per pack – i.e. *T_E_* plus I$ 1.00 – and T_VAT_*  is the new VAT amount per pack. Total excise revenue was calculated as the excise per pack multiplied by the quantity of cigarette packs sold in the retail market (*S*).

### Consumption and use

The extent to which higher cigarette prices reduce consumption is governed by the price elasticity of the demand. For example, a price elasticity of −0.3 means that a 10% increase in cigarette prices will reduce cigarette consumption by 3%. Studies in high-income countries have revealed price elasticities that range from −0.25 to −0.5 while studies in low- and middle-income countries have revealed corresponding elasticities between −0.2 and −0.8.^22^ It appears that cigarette consumers in low- and middle-income countries are generally more price-sensitive that their counterparts in high-income countries. In this study, the price elasticities of cigarettes in high-, middle- and low-income countries were assumed to be −0.3, −0.4 and −0.5, respectively. The number of packs sold in response to the price increase (S*) was calculated as: *S* × (1 + Δ*P* × ε*_p_*) (3) where Δ*P* is the percentage change in the retail price and ε*_p_* is the price elasticity of demand. As the elasticities are short-term parameters, we assumed that the full impact of the price increase on consumption would occur within one to three years. Table 1 shows the key assumptions used in the modelling.

**Table 1:** Summary of key model assumptions and parameters

Variable	Country income group			
	Low	Lower- middle	Upper- middle	High
**Tax and market parameters**^a^				
Increase in excise, I$/20-cigarette pack^b^	1.00	1.00	1.00	1.00
Pass through of taxes onto price, %	100	100	100	100
Increase in industry margins per pack, %	2.5	2.5	2.5	2.5
**Short-term time horizon**^c^				
Elasticity in cigarette price	−0.50	−0.40	−0.40	−0.30
Elasticity in prevalence of smoking	−0.25	−0.20	−0.20	−0.15
**Long-term time horizon**^d^				
Risk of a smoking-attributable death, %^e^	33	33	33	33
Mortality adjustment for quitters, %^f^	67	67	67	67

I$: international dollars.

^a^ In effect in the 12 months following the increase in excise, in 2014.

^b^ International dollars were based on the purchasing power parity exchange rates for 2014.

^c^ In effect one to three years after the increase in excise.

^d^ In effect from the end of the third year after the increase in excise.

^e^ Among the adult daily smokers who were alive in 2014.

^f^ The probability that an adult daily smoker in 2014 – who would have died from smoking – will avoid a smoking-attributable death by quitting.

The price elasticity of demand reflects a combination of conditional demand – i.e. the amount or intensity of smoking – and smoking prevalence.^23^ Global evidence suggests that, for cigarettes, half of the impact of higher prices comes from a reduction in smoking prevalence.^22^^–^^24^ Consequently, for our model, we assumed that the prevalence elasticity was half of the price elasticity – i.e. −0.15, −0.2 and −0.25 in high-, middle- and low-income countries, respectively. We used these prevalence elasticities to estimate the reduction in the number of smokers in the current adult population that would result from our modelled increase in excise. The prevalence elasticities we used are the same as those previously used to assess the global impact of tobacco control policies.^25^

### Public health outcomes

We used a single cohort approach^23^^,^^25^ to measure the impact of tobacco taxation on the expected number of smoking-attributable deaths among the world’s adults who were alive in 2014. In this approach the impact of tobacco control policies was measured first in terms of the reduction in the number of smokers among the current adult population and then in terms of the future health outcomes for the same population cohort over the course of their remaining lives. We defined anyone older than 15 years as an adult and we used a medium to long-term time horizon to cover the remaining lives of the current cohort of adult smokers. For each study country, we estimated the baseline number of adult daily cigarette smokers from the size of the adult population and the prevalence of daily cigarette smokers among the adults.

Epidemiological studies over the past 50 years have shown that tobacco ultimately kills a third to half of all people who use it.^26^^,^^27^ By applying a relatively low risk of a smoking-attributable death – of 33% – to the adult daily smokers in our model, we aimed to produce a conservative estimate of the number of smoking-attributable deaths that could be averted by the tax intervention.

We estimated the positive impact of tobacco taxation on health as the expected decrease in the number of smoking-attributable deaths – after accounting for those current smokers who will cease smoking before they die. The benefits of quitting are many and occur early for several serious diseases.^28^ Overall, adults who cease smoking before they reach middle age avoid almost all the excess hazards of smoking.^26^ Nonetheless, some adjustment is required to account for the fact that not all smokers who quit can avoid early death. National studies typically use a mortality adjustment factor of 70% for smokers who quit.^29^^,^^30^ A global mortality adjustment factor has been calculated on the assumption that 95%, 75%, 70%, 50% and 10% of those who cease smoking when aged 15 to 29, 30 to 39, 40 to 49, 50 to 59 and at least 60 years, respectively, will avoid an early death.^23^ We applied the same percentages to the age profile of the world’s population in 2014, leading to a mean adjustment factor of 67%. Thus, we assumed that 67% of the adult daily smokers in 2014 who would otherwise have suffered an early death from a disease caused by smoking would avoid such a death if they ceased smoking. We estimated the number of smoking-attributable deaths averted as a result of the tax increase as 67% of 33% of the reduction in the number of daily adult smokers resulting from the increase in cigarette prices.

## Results

### 2014 baseline

In 2014, the mean amount of excise was estimated to be I$ 1.37 per 20-cigarette pack. This represented 43% of the mean retail price of I$ 3.20 per pack (Table 2). As the total annual cigarette consumption was calculated to be 294 billion packs, the total excise revenue generated globally from the sale of cigarettes was estimated to be I$ 402 billion – or about 328 billion United States dollars (US$).

**Table 2:** Simulation model of the cigarette market by country income group, 181 countries

Variable	Country income group				
	Low	Lower- middle	Upper- middle	High	All
**Excise**					
2014 baseline value, I$/20-cigarette pack	0.80	1.10	0.94	2.53	1.37
After simulated increase, I$/20-cigarette pack	1.82	2.16	1.99	3.59	2.46
Change, %	+127	+96	+111	+42	+80
**Retail price**					
2014 baseline value, I$/20-cigarette pack	2.02	2.42	2.62	5.07	3.20
After simulated excise increase, I$/20-cigarette pack	3.30	3.69	3.90	6.35	4.55
Change, %	+63	+53	+48	+25	+42
**Annual consumption**					
2014 baseline value, millions of 20-cigarette packs	6878	48 938	163 440	74 447	293 704
After simulated excise increase, millions of 20-cigarette packs	4656	37 645	130 138	68 052	240 491
Change, millions of 20-cigarette packs (%)	−2222 (−32)	−11 293 (−23)	−33 302 (−20)	−6395 (−9)	−53 212 (−18)
**Annual excise revenue**					
2014 baseline value, millions of I$	5520	54 020	154 155	188 477	402 172
After simulated excise increase, millions of I$	8492	81 275	258 782	244 052	592 600
Change, millions of I$ (%)	+2971 (+54)	+27 256 (+50)	+104 627 (+68)	+55 574 (+29)	+190 428 (+47)
**Prevalence of daily cigarette smoking**					
2014 baseline value, % of adults	9.1	9.3	17.5	18.2	14.1
After simulated excise increase, % of adults	7.7	8.3	15.8	17.5	12.9
Change, %	−16	−11	−10	−4	−9
**No. of adult daily smokers (thousands)**					
2014 baseline value	44 584	165 037	337 715	192 936	740 271
After simulated excise increase	37 448	147 025	304 910	185 272	674 654
Change	−7136	−18 012	−32 805	−7664	−65 617
**No. of smoking-attributable deaths (thousands)**					
Predicted from 2014 baseline data	14 861	55 012	112 572	64 312	246 757
After simulated excise increase	13 275	51 010	105 282	62 609	232 175
Change	−1586	−4003	−7290	−1703	−14 582

I$: international dollars.

Note: International dollars were based on the purchasing power parity exchange rates for 2014.

Although we estimated that there were 740 million adults who were daily cigarette smokers worldwide in 2014, almost 320 million (43%) of these smokers lived in just four middle-income countries: Brazil, China, India and the Russian Federation. These numbers exclude smokers of other forms of tobacco – e.g. bidi smokers in south-east Asia. We estimated that, under the baseline scenario, at least 247 million daily smokers from among the adult population in 2014 will ultimately die from a smoking-attributable disease.

### Tax simulation

Raising excise by I$ 1.00 per 20-cigarette pack in all countries would generate a substantial increase in cigarette tax yields in all countries. Excise per pack would increase by 80% globally (Table 2). Tax yields would increase the most in the Eastern Mediterranean – partly because many countries in this region did not levy any cigarette excise in 2014 (Table 3). The mean retail price of cigarettes would increase by 42% globally. Cigarette prices would increase by a mean of 63% in low-income countries but only by a mean of 25% in high-income countries (Table 2).

**Table 3:** Simulation model of the cigarette market by WHO region, 181 countries

Variable	WHO region					
	Africa	Americas	Eastern Mediterranean	Europe	South-East Asia	Western Pacific
**Excise**						
2014 baseline value, I$/20-cigarette pack	0.74	2.36	0.77	2.51	1.40	0.87
After simulated increase, I$/20-cigarette pack	1.80	3.42	1.88	3.62	2.43	1.90
Change, %	+143	+45	+143	+44	+73	+118
**Retail price**						
2014 baseline value, I$/20-cigarette pack	2.72	5.46	2.06	4.60	2.91	2.50
After simulated excise increase, I$/20-cigarette pack	4.09	6.77	3.43	5.99	4.13	3.75
Change, %	+51	+24	+66	+30	+42	+50
**Annual consumption**						
2014 baseline value, millions of 20-cigarette packs	6 917	26 086	17 486	57 899	28 858	156 457
After simulated excise increase, millions of 20-cigarette packs	5 253	23 614	12 280	51 102	23 640	124 603
Change, millions of 20-cigarette packs (%)	−1 664 (−24)	−2 472 (−9)	−5 207 (−30)	−6 797 (−12)	−5 218 (−18)	−31 855 (−20)
**Annual excise revenue**						
2014 baseline value, millions of I$	5 110	61 588	13 520	145 447	40 418	136 089
After simulated excise increase, millions of I$	9 444	80 857	23 092	185 043	57 409	236 756
Change, millions of I$ (%)	4 333 (+85)	19 269 (+31)	9 572 (+71)	39 596 (+27)	16 991 (+42)	100 668 (+74)
**Prevalence of daily cigarette smoking**						
2014 baseline value, % of adults	7.8	11.2	11.6	21.1	8.6	19.9
After simulated excise increase, % of adults	6.8	10.6	9.8	20.0	7.8	17.9
Change, %	−12	−5	−15	−5	−9	−10
**No. of adult daily smokers (thousands)**						
2014 baseline value	41 535	81 221	46 341	157 913	113 650	299 611
After simulated excise increase	36 407	76 862	39 399	149 645	103 419	268 922
Change	−5 127	−4 359	−6 942	−8 268	−10 232	−30 689
**No. of smoking-attributable deaths (thousands)**						
Predicted from 2014 baseline data	13 845	27 074	15 447	52 638	37 883	99 870
After simulated excise increase	12 705	26 105	13 904	50 800	35 610	93 051
Change	−1 139	−969	−1 543	−1 837	−2 274	−6 820

I$: international dollars; WHO: World Health Organization.

Note: International dollars were based on the *purchasing power parity *exchange rates for 2014.

Global cigarette consumption would decrease by 18% – representing 53 billion fewer cigarette packs compared with 2014 (Table 2). Cigarette consumption would decline most in the Western Pacific – reflecting this region’s large consumption base. The amount of cigarette excise revenue generated throughout the world would increase by I$ 190 billion – or about US$ 141 billion. All income groups and regions would see substantial growth in excise revenues. The African continent would expand excise revenue from cigarettes by as much as 85% (Table 3).

**Fig. 1:**
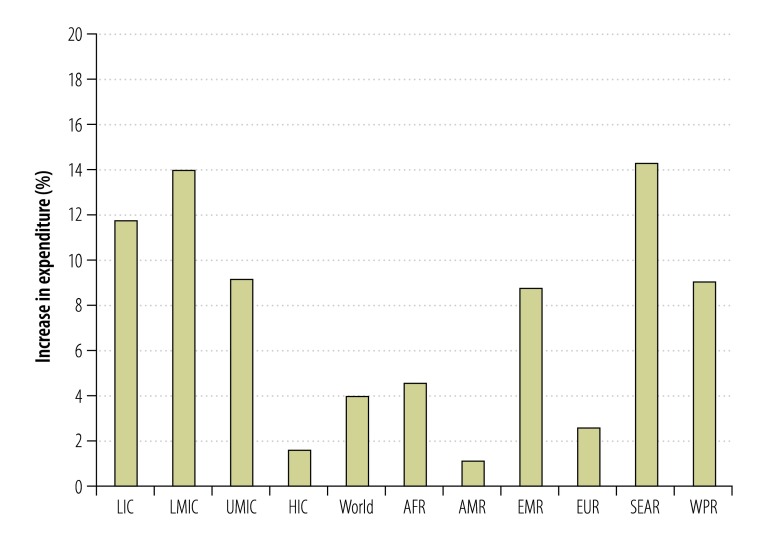
Increases in government health expenditures resulting from the simulated increase in excise on cigarettes, 181 countries

AFR: African Region; AMR: Region of the Americas; EMR: Eastern Mediterranean Region; EUR: European Region; HIC: high-income country; LIC: low-income country; LMIC: lower-middle-income country; SEAR: South-East Asia Region; UMIC: upper-middle-income country; WPR: Western Pacific. Region.

Note: Excise is a tax imposed on selected commodities and is the main fiscal instrument that governments use to generate extra tax revenue from those commodities.

The extra excise revenue from cigarettes would help create the fiscal space needed by countries to meet their development priorities. For example, if all of the extra revenue from raising cigarette excise was allocated to government health budgets, then public expenditure on health could increase by 4% globally (Fig. 1). A third of all low- and middle-income countries would be able to increase public health expenditure by more than 10% in this manner.

**Fig. 2:**
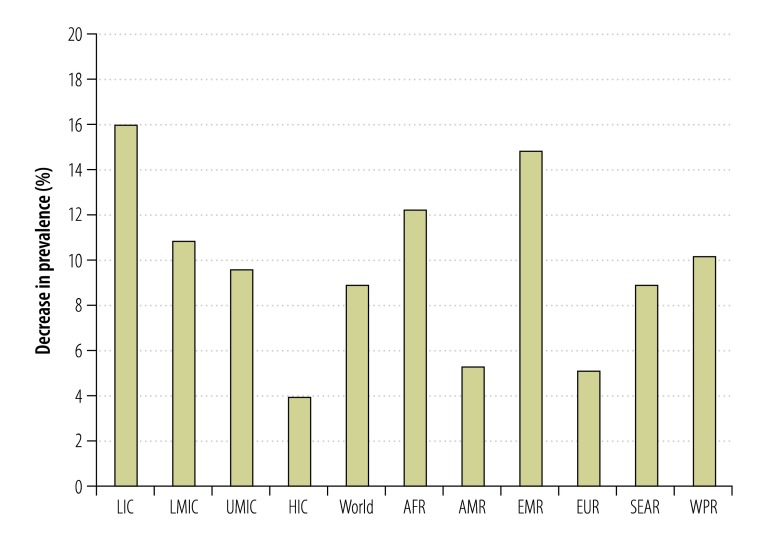
Decreases in prevalence of daily cigarette smoking among adults resulting from the simulated increase in excise on cigarettes, 181 countries

AFR: African Region; AMR: Region of the Americas; EMR: Eastern Mediterranean Region; EUR: European Region; HIC: high-income country; LIC: low-income country; LMIC: lower-middle-income country; SEAR: South-East Asia Region; UMIC: upper-middle-income country; WPR: Western Pacific. Region.

Note: Excise is a tax imposed on selected commodities and is the main fiscal instrument that governments use to generate extra tax revenue from those commodities.

In terms of health outcomes, the prevalence of daily cigarette smoking among adults would decline by 9% in relative terms – i.e. from 14.1% to 12.9% of the adult population (Fig. 2). This decrease translates into 66 million fewer smokers. The expected number of smoking-attributable deaths from among the world’s adult population in 2014 would decrease by 15 million – reflecting a decline of about 6% in smoking-related mortality among this cohort (Table 2). The majority of the smoking-attributable deaths averted would be in low- and middle-income countries.

## Discussion

WHO has been working with its Member States to implement the FCTC. For example, it has been collaborating with ministries of finance to help them adopt better policies on tobacco taxation. Detailed, country-level tax models – similar to the one described here – have helped to frame discussions on the policy objectives of tobacco taxation. A frequent and important precondition, from the perspective of public finance, is the need for reforms to generate higher tax revenues sustainably – at least over the short to medium term. Among the other concerns of government officials that are being addressed in these collaborations is the threat of illicit trade. In this present study, we do not address illicit trade directly. However, between-country differences in cigarette taxes and prices would be narrowed – not widened – by the tax increase that we modelled. This might be expected to reduce the incentive for illicit trade.

In reality, illicit trade occurs in low-tax jurisdictions as well as high-tax ones and there is no direct correlation between rates of tobacco taxation and tobacco smuggling.^31^ Factors other than taxes and prices serve to motivate or enable illicit trade. The administrative capacity of many tax and customs departments needs to be strengthened.^22^^,^^31^ Given the transnational nature of the illicit trade in cigarettes, it is clear that a coordinated international response is needed. In November 2012, the Conference of Parties to the FCTC adopted the Protocol to Eliminate Illicit Trade in Tobacco Products.^32^ While negotiating this protocol, Member States have agreed to a set of control measures that should help address the critical administration and transnational issues.

The single cohort approach that we used in this study fails to incorporate the dynamic aspects of changing demographics and smoking prevalence. These considerations require the use of a structural model such as that used in the global burden of disease projections.^33^^,^^34^ However, structural models are relatively sophisticated and data-intensive and beyond the intended scale of our study. In addition, when another study compared their single cohort analysis with the results of using dynamic models in nine countries, they found that the dynamic aspects of policy change did not substantially change their main findings.^25^

Another limitation of the present study is that we applied the same mortality risk in all countries. The relative risks have been found to be lower in low- and middle-income countries than in high-income ones^25^ – possibly because of population differences in age at initiation of smoking, smoking intensity and/or the background risk from other causes of death. Structural models have included smoking impact factors that indirectly measure the accumulated risk. Some studies report sensitivity analyses based on a range of 33% to 50% mortality risk.^25^^,^^30^ In this study we applied conservative assumptions to ensure that the results were also conservative. Therefore, our estimate of the number of smoking-attributable deaths averted could well be an underestimate – especially in Europe and North America where the tobacco epidemic is currently strongest.

## Conclusion

Tobacco taxation can prevent millions of smoking-attributable deaths throughout the world and contribute to the achievement of global health objectives, such as SDG target 3.4. There is substantial potential for tobacco taxation to create the fiscal space needed to finance development, particularly in low- and middle-income countries.
